# Supplementary figures and images for: Development and Comprehensive Characteristics of Thermosensitive Liquid Suppositories of Metoprolol Based on Poly(lactide-co-glycolide) Nanoparticles
Source: Int J Mol Sci. 2022 Nov 8;23(22):13743. doi: 10.3390/ijms232213743 (PMC9693477; doi:10.3390/ijms232213743)

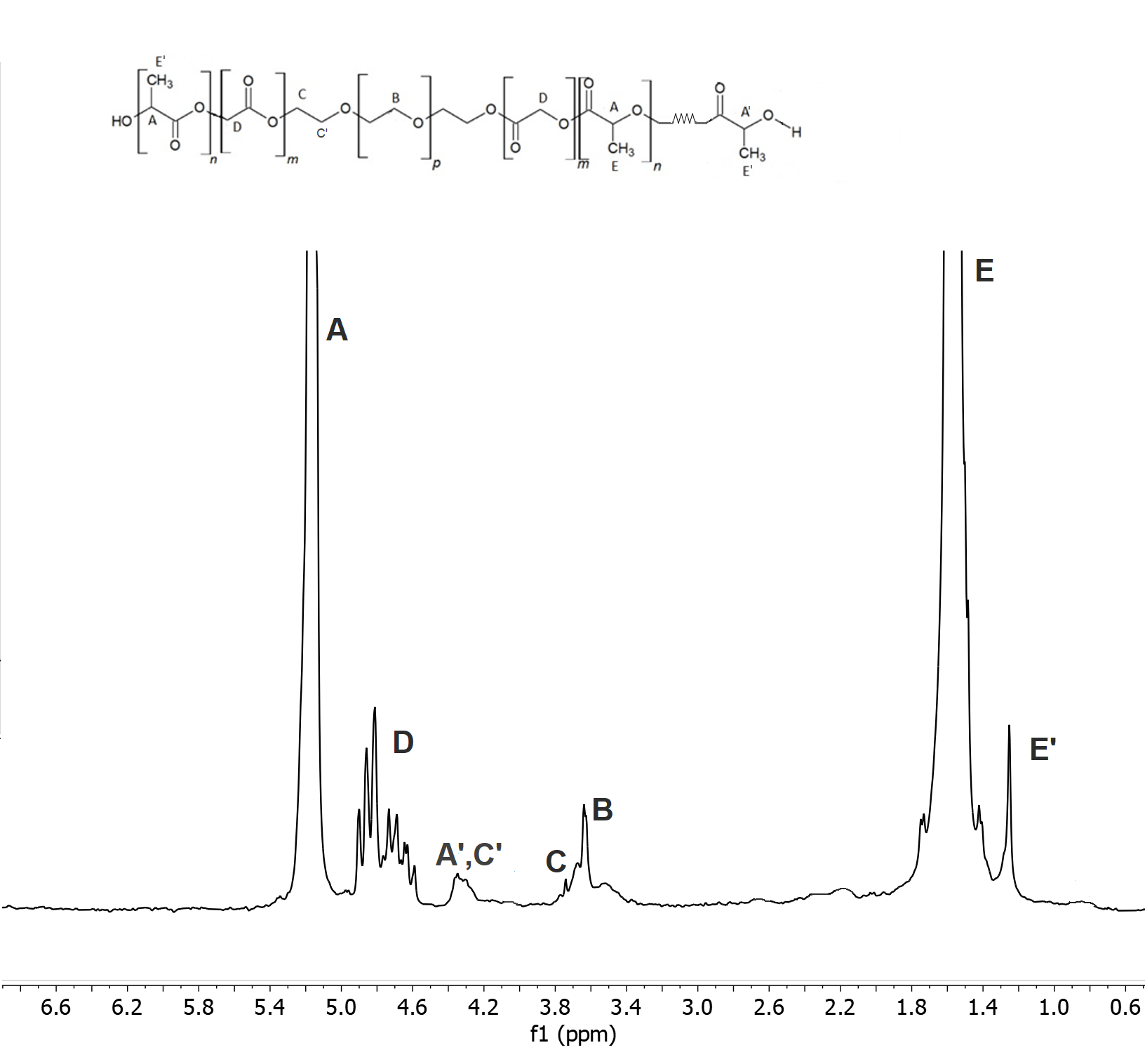

Supplement: Supplementary file 1 [file ijms-23-13743-s001.zip › Figure S1.tif]

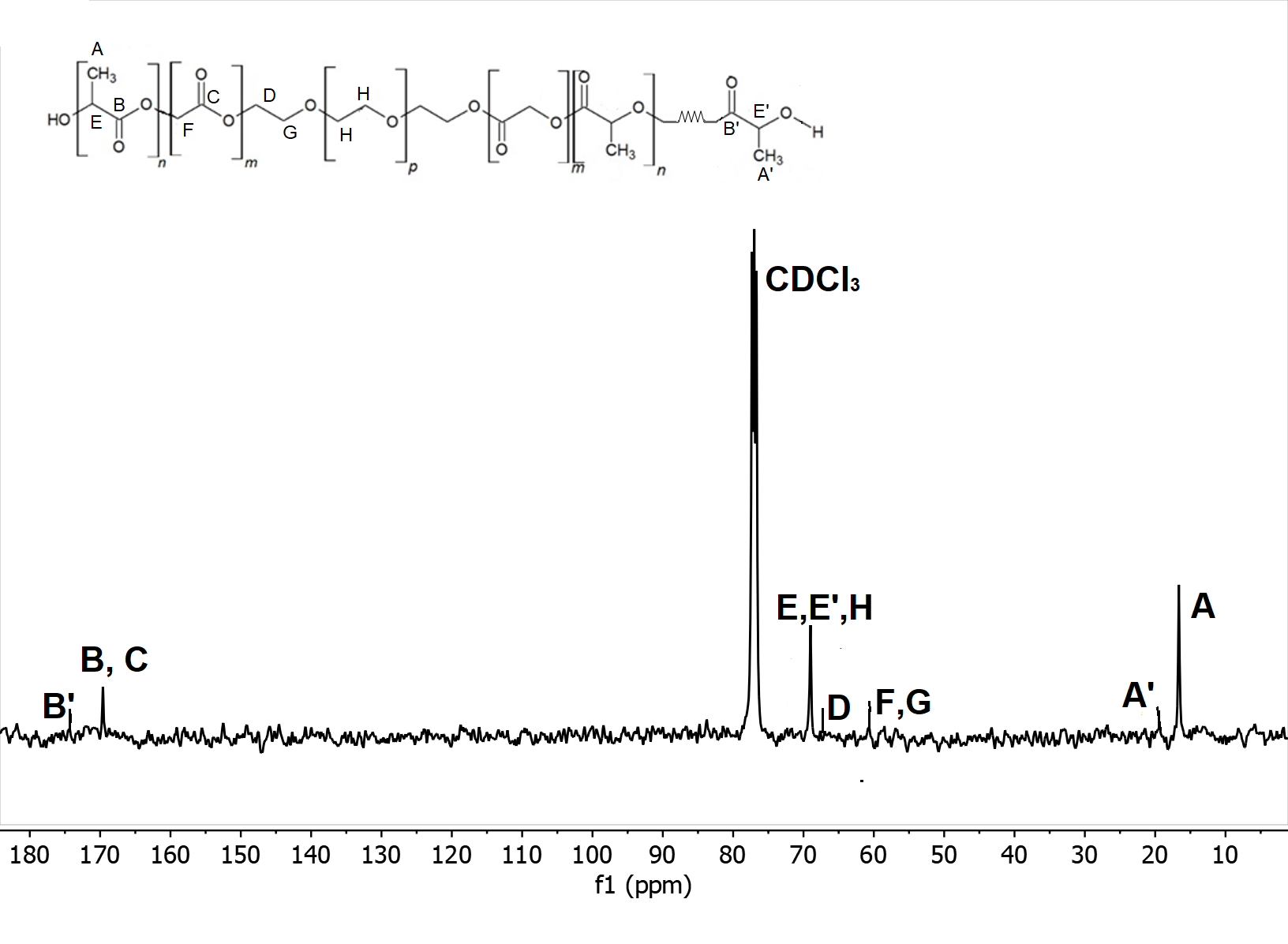

Supplement: Supplementary file 1 [file ijms-23-13743-s001.zip › Figure S2.tif]

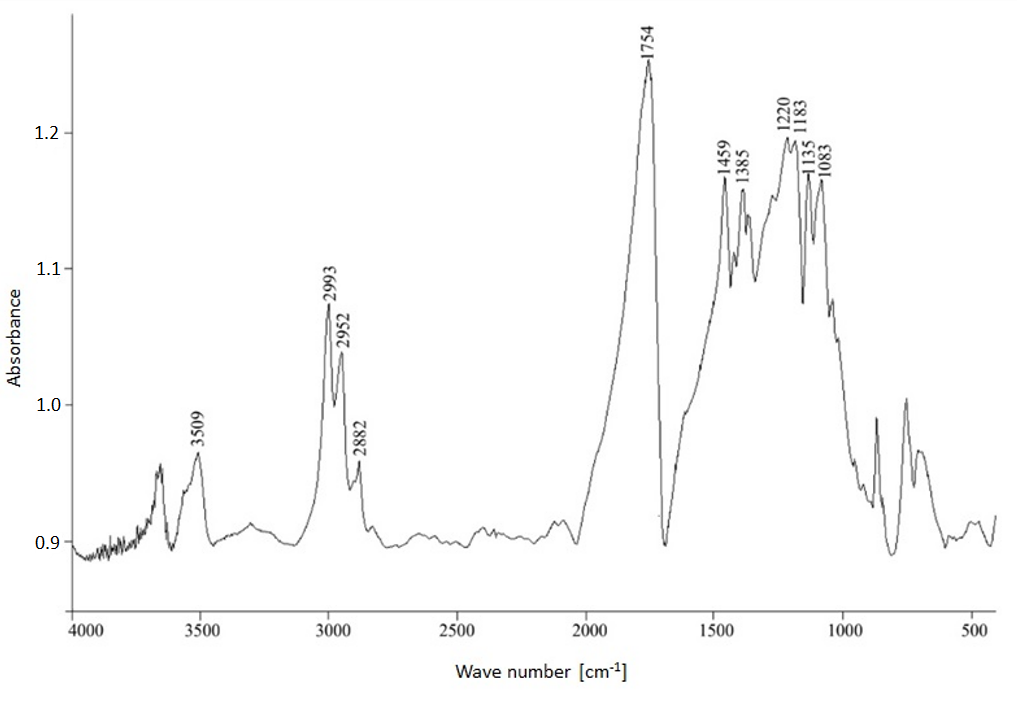

Supplement: Supplementary file 1 [file ijms-23-13743-s001.zip › Figure S3.tif]

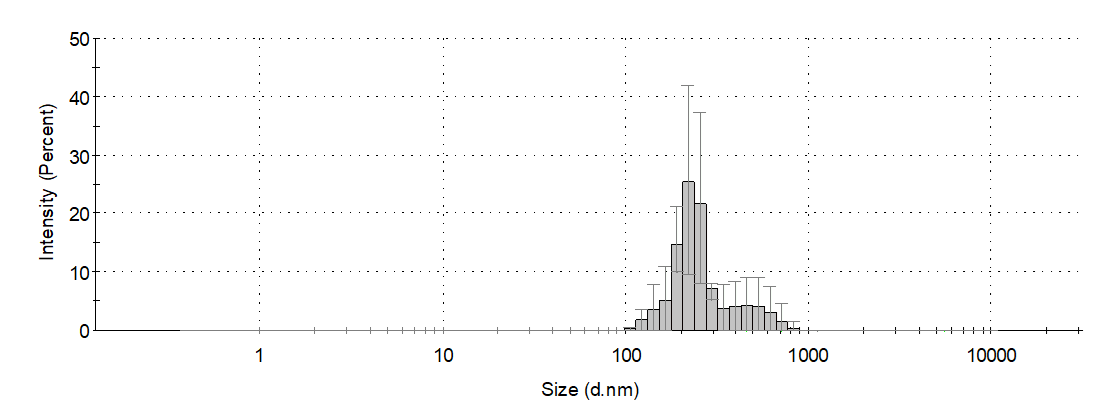

Supplement: Supplementary file 1 [file ijms-23-13743-s001.zip › Figure S4.tif]

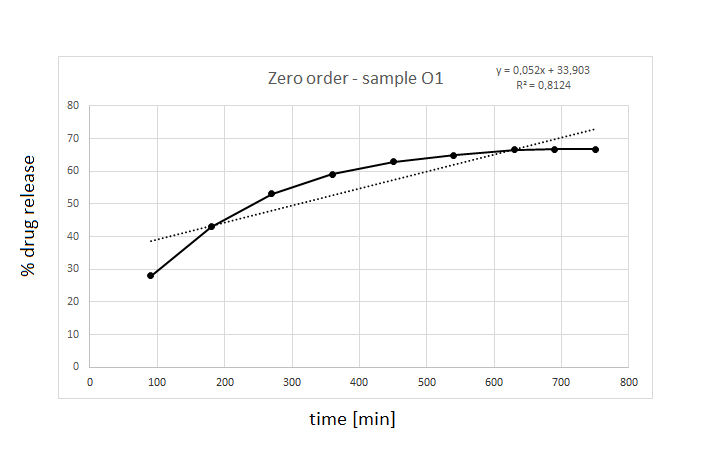

Supplement: Supplementary file 1 [file ijms-23-13743-s001.zip › Figure S5.tif]

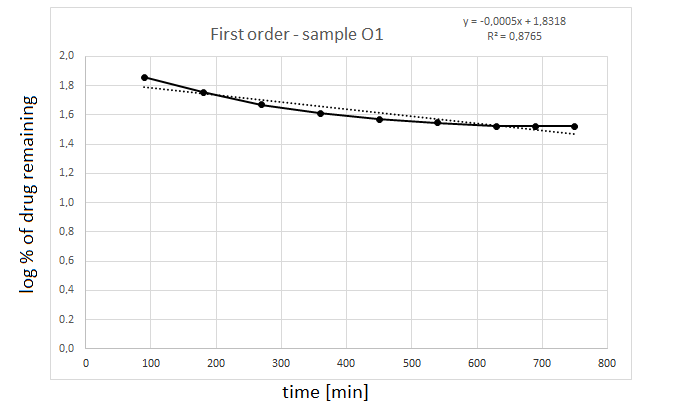

Supplement: Supplementary file 1 [file ijms-23-13743-s001.zip › Figure S6.tif]

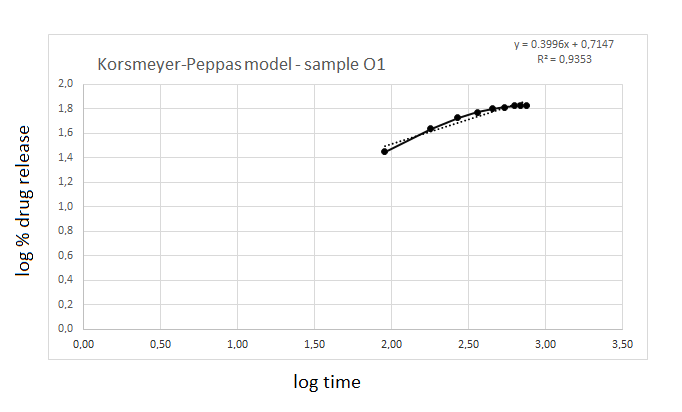

Supplement: Supplementary file 1 [file ijms-23-13743-s001.zip › Figure S7.tif]

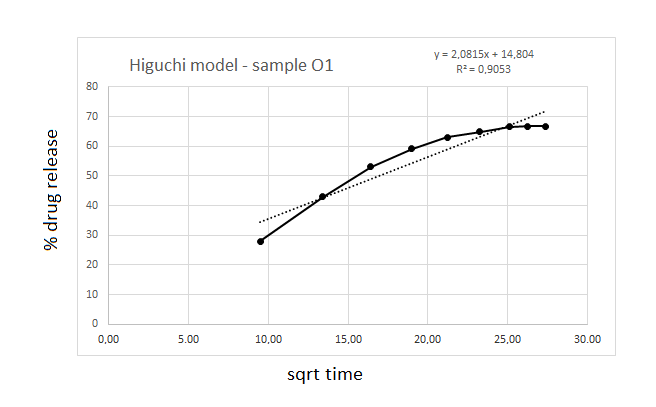

Supplement: Supplementary file 1 [file ijms-23-13743-s001.zip › Figure S8.tif]

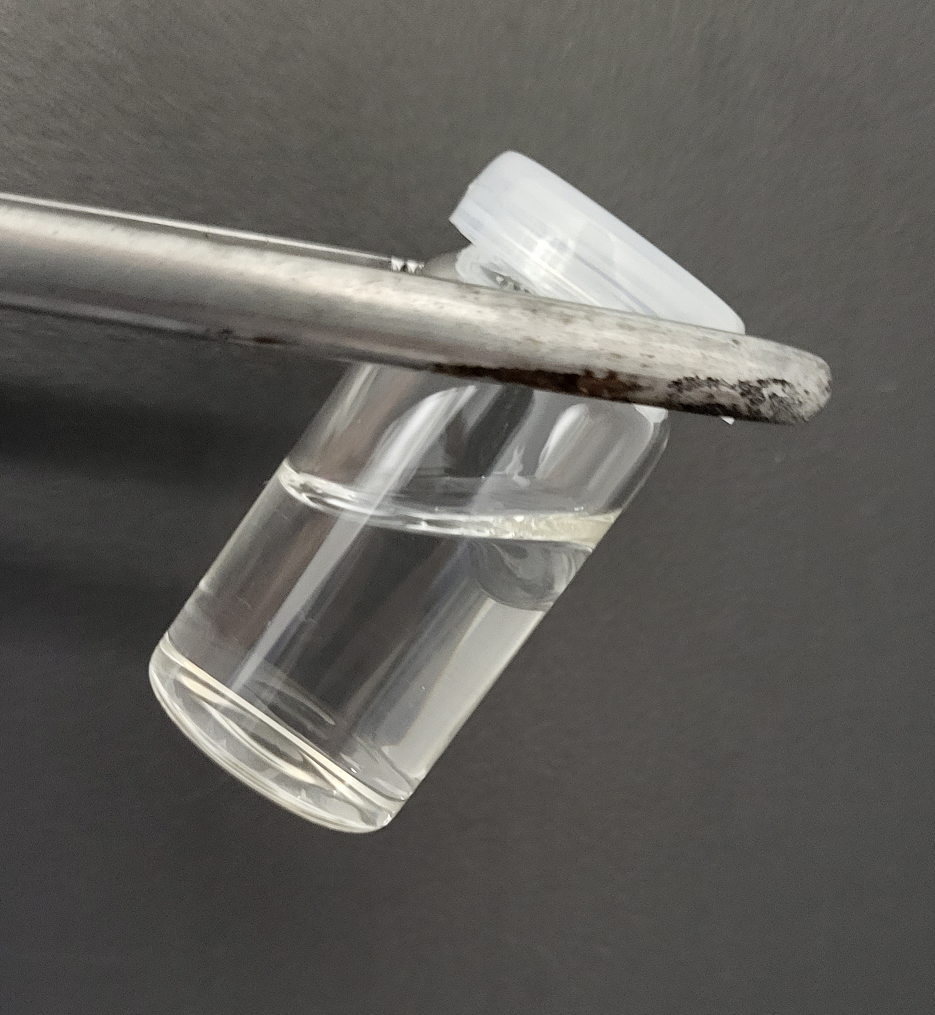

Supplement: Supplementary file 1 [file ijms-23-13743-s001.zip › Figure S9.tif]
